# Pre-pandemic patterns in colorectal cancer mortality and Black-White inequities across the 30 most populous US cities

**DOI:** 10.3389/fepid.2025.1681088

**Published:** 2025-10-13

**Authors:** Maryam Bolouri, Nazia S. Sayed, Maureen R. Benjamins, Nicholas R. Munoz, Tyler Halterman, Abigail Silva

**Affiliations:** ^1^ Stritch School of Medicine, Loyola University Chicago, Maywood, IL, United States; ^2^ Sinai Urban Health Institute, Sinai Chicago, Chicago, IL, United States; ^3^ Department of Internal Medicine, UHS Southern California Medical Education Consortium, Temecula, CA, United States,; ^4^ Parkinson School of Health Sciences and Public Health, Loyola University Chicago, Maywood, IL, United States, United States

**Keywords:** colorectal cancer, cancer mortality, public health, health equity, cancer

## Abstract

**Background:**

Colorectal cancer (CRC) is a leading cause of cancer deaths in the United States. While national CRC mortality rates have improved over time, this rate differs between non-Hispanic (nH) Black and nH White populations and by geography.

**Methods:**

The 30 most populous cities in the US were analyzed using national vital statistics data. Numerators were obtained from death certificates of residents of these cities with CRC as the underlying cause of death. US Census data provided population-based denominators. We calculated Black:White rate ratios (RRs) and corresponding confidence intervals for the most recent time period studied (2017–2019) to assess racial inequities. We calculated average annual percent changes to evaluate CRC mortality trends from 2009–2019.

**Results:**

CRC mortality rates statistically significantly decreased nationally and in 25 of the 30 cities. In most cities, nH Black populations experienced a greater decrease in CRC mortality over time than nH White populations. However, in 20 cities, the Black:White CRC mortality rate ratio was greater than 1 (ranging from 1.28 in New York to 2.68 in Washington, D.C.; *p* < 0.05), indicating persistent racial inequities. Between 2009 and 2019, six cities saw statistically significant decreases in racial inequities, two cities saw increases, and the remaining cities demonstrated persistent disparities.

**Conclusions:**

Despite improvements in CRC mortality, Black:White disparities persist. Structural racism may contribute to these disparities through differential access to care and risk factor exposure. Identifying geographic differences in Black:White CRC mortality may serve as a catalyst for local governments to implement place-based initiatives that reduce screening barriers and contribute to health equity.

## Introduction

In the United States (US), colorectal cancer (CRC) is the third most common cancer diagnosed and the second leading cause of cancer deaths, with 52,900 Americans expected to die of CRC in 2025 (1). CRC mortality rates have improved considerably in the US over the last few decades due to a combination of screening and improved treatment, with CRC mortality decreasing by 55% in men since 1978, and 61% in women since 1969 (2–4). However, the improvement in CRC mortality has not been experienced equitably, as considerable racial disparities persist. There is evidence of a widening CRC disparity in incidence and mortality between non-Hispanic (nH) Black and nH White Americans since the 1980s (5, 6). The CRC incidence rate (per 100,000) is 17% higher for nH Black (41.7) vs. nH White Americans (35.7). The CRC mortality gap is even wider, as the most recent data show that the CRC mortality rate (per 100,000) for the nH Black population (17.6) is 34% higher than for the nH White population (13.1) (7).

Racial disparities in CRC mortality differ according to geographic location (8, 9). A recent report showed that among the 41 states for which both the nH Black and nH White rates were available, the nH Black rate was (1%–66%) higher than the nH White rate in 39 states (10). At a smaller geographic level, one study found that most US counties (61%) had persistent or worsening racial disparities in CRC mortality rates while less than 5% of counties had achieved and sustained equal rates (8). However, little information on (overall or race-specific) CRC mortality trends at the city level is available, although some studies suggest that racial disparities in CRC mortality from other types of cancers vary dramatically at this geographic unit (11–13). Additionally, CRC burden and screening patterns vary between large urban centers and rural areas, with rural populations generally experiencing lower screening rates, later-stage diagnoses, and higher mortality (14). While several important initiatives, including the City Health Dashboard and Big Cities Health Inventory, provide city-level data on CRC mortality and racial disparities, these resources do not assess CRC mortality trends in Black:White inequities (15, 16). Because local policy change generally happens at the city level, local data is needed to inform place-based initiatives. Additionally, as a growing number of cities commit to addressing health inequities, race-specific estimates and an explicit assessment of trends in racial disparities are needed to inform and evaluate efforts (15, 17, 18). Furthermore, assessing CRC mortality during the decade prior to the COVID-19 pandemic provides a point of comparison for future insight on how cities may have recovered from the pandemic, when cancer screening was negatively impacted and recovery varied between metropolitan and non-metropolitan areas (19, 20).

To address this critical data gap, the current study provides a detailed assessment on CRC mortality trends at the city level. We calculate and compare changes in overall and race-specific CRC mortality rates as well as Black:White inequity in rates. Population-level research on breast and colorectal cancer in local settings has led to improvements in policies and programs (13, 21, 22). A more comprehensive documentation of CRC rates across large cities can be used by government agencies, policy makers, and community leaders to address modifiable factors and reduce CRC mortality and racial inequities.

## Methods

This serial cross-sectional study used data from the National Vital Statistics System (NVSS) and Census Bureau's American Community Survey (ACS). The manuscript adhered to the Strengthening the Reporting of Observational Studies in Epidemiology (STROBE) guidelines for cross-sectional studies. This study, which uses de-identified data, was deemed exempt from review by the Mount Sinai Hospital institutional review board.

### Study population

We identified the 30 most populous cities, which comprise 12.5% of the US population, based on 2014 US Census Bureau data. Inclusion was limited to these cities to ensure an adequate number of total and race-specific deaths for city-level analyses. These cities are also part of the Big Cities Coalition (16). Due to questions about the geographic area considered to be part of Las Vegas in the mortality and census data files, we substituted it with Milwaukee which was the 31st most populous city. Of the 30 cities, county data were used for three cities (Louisville and Jefferson County, KY; Nashville and Davidson County, TN; Indianapolis and Marion County, IN) where the city and county have distinct geographical boundaries but have formed a consolidated government.

### Data sources

Mortality data came from the NVSS Multiple Cause of Death data files for 2009–2019 (23). The NVSS provides official mortality data from death certificates for all causes of death across states and cities based on place of residence.

Resident deaths that occurred in 2009–2019 were included if the underlying cause of death on the death certificate was colorectal cancer (ICD-10 Codes: C18–C21, C26.0) in accordance with the Healthy People Initiative (24). Deaths were excluded if they were among non-US residents or were missing age. A total of 29,210,014 death records were assessed for eligibility, from which 57,844 records of non-residents of the US were excluded as is standard with published mortality statistics reports (25). In addition, 1,652 records where age was missing were excluded as that is information needed for age-adjustment. Of the remaining records, we excluded 28,567,483 where the cause of death was not colorectal cancer, leaving 583,035 death records included in the analysis.

Population denominator data for the total, nH White (White), and total Black populations were obtained from the ACS 5-year estimates. The ACS five-year estimate includes data collected over a 60-month period. For instance, the 2019 ACS five-year estimate is an average of the data collected between 2015 and 2019. These multiyear-based estimates increase the statistical reliability of the data for smaller geographic areas and small population subgroups (26).

Data for the nH Black population are most recently available in the 2010 Decennial Census. Using 2010 Census data, we divided the nH Black population by the total (Hispanic and non-Hispanic) Black population to find the proportion of the Black population that was non-Hispanic in each city and age group. Then, we applied these city- and age-specific proportions to the respective five-year ACS estimates of the total Black population.

### Measures

Age-adjusted total and race-specific CRC mortality rates (per 100,000 population) were calculated for the US and each of the 30 cities (27). For 2009–2019, we extracted all race-specific CRC deaths by age group (i.e., 0–4, 5–14, 15–24, 25–34, 35–44, 45–54, 55–64, 65–74, 75–84, and 85 years and older) and place of residence. Three-year estimates were used to ensure sufficient deaths per city. We calculated three-year age-adjusted CRC mortality rates using the US 2000 standard population. The population at risk for each three-year time period of analysis was estimated by applying a multiplier of 3 to the ACS and Census-derived population estimates. As an example, for the 2017–2019 population at risk, we used the ACS 5-year estimate for 2018 and applied a multiplier of 3 to estimate the population during the entire period. For the sake of brevity, non-Hispanic Black will henceforth be referred to as “Black” and non-Hispanic White as “White.” To assess the relative racial inequity, we calculated Black:White rate ratios (RRs) by dividing the Black-specific age-adjusted CRC mortality rate by the respective White-specific rate. Measures were suppressed in instances where cities had <20 CRC deaths for the overall, White, or Black populations during a three-year period as the estimates are considered unreliable (27).

For the trend analyses, three-year rolling averages were used as a smoothing technique. For instance, the age-adjusted three-year average CRC mortality rate for 2018 is the average of 2017, 2018, and 2019. Therefore, the 11 years of annual data (2009–2019) produces nine three-year rolling average data points.

### Statistical analysis

Black:White rate ratios and trends in those ratios were estimated for the US and each city. The rate ratios for the most recent time period (2017–2019) were calculated along with their standard errors and 95% confidence intervals (CI) using a Taylor series expansion technique (28). Trends were examined for each city using log linear joinpoint regression models to calculate the average annual percentage changes (AAPCs) and their 95% CIs (29, 30). To evaluate inequities, we imported the annual rate ratios and their standard errors to calculate the AAPCs. The AAPC is the weighted average of the annual percentage change (APCs) from the joinpoint model where the weights equal the length of the APC interval. This approach provides a more stable estimate of the trend within a fixed interval (29). The AAPC helps determine the direction, magnitude, and significance of changes in rates and rate ratios over time. An increase is denoted by an AAPC >0 (*p* < 0.05) and a decrease by an AAPC < 0 (*p* < 0.05); otherwise, the trend is considered stable. All statistical tests were two-sided. Statistical analysis was conducted using SAS version 9.4 and Joinpoint software version 4.9.0.1 (https://surveillance.cancer.gov/joinpoint/).

## Results

At the most recent time point (2017–2019), the CRC mortality rate for the US was 14 per 100,000 individuals (Table 1). Across cities, the overall rate ranged from 10 (Boston) to 19 (Memphis). The CRC mortality rate among Black US residents was 19 and ranged from a low of 12 (Boston) to a high of 27 (Houston). The CRC mortality rate among White US residents was 14 and ranged from 7 (Washington, DC) to 17 (San Antonio). During this time, the CRC mortality rate among the US Black population was 38% higher than among the White population [rate ratio (RR) = 1.38; 95% CI: 1.36–1.40]. The Black rate was statistically significantly higher than the White rate in 20 cities, with rate ratios ranging from 1.28 in New York (95% CI: 1.18–1.39) to 2.69 in Washington, DC (95% CI: 1.96–3.68). Among the remaining cities, there were no statistically significant differences between the Black and White population rates.

**Table 1:** Colorectal cancer mortality rates and Black-White mortality rate ratios for the United States and 30 most populous cities, 2017–2019.

Colorectal Cancer Mortality Rate Per 100,000				
City, State	Total	Non- Hispanic Black	Non- Hispanic White	Black-White Rate Ratio (95% CI)
United States	14.04	19.43	14.03	1.38 (1.36, 1.41)
Austin, TX	11.96	20.00	10.84	1.84 (1.27, 2.68)
Baltimore, MD	18.32	19.96	16.82	1.19 (0.94, 1.50)
Boston, MA	10.08	12.93	9.87	1.31 (0.94, 1.83)
Charlotte, NC	10.28	13.00	9.09	1.43 (1.07, 1.91)
Chicago, IL	16.18	23.68	13.29	1.78 (1.57, 2.02)
Columbus, OH	13.88	16.24	14.21	1.14 (0.89, 1.47)
Dallas, TX	15.20	23.62	14.02	1.69 (1.39, 2.05)
Denver, CO	13.51	22.14	11.51	1.92 (1.36, 2.71)
Detroit, MI	17.98	19.25	14.69	1.31 (0.92, 1.87)
El Paso, TX	11.80	—	13.24	—
Fort Worth, TX	14.56	21.45	14.25	1.51 (1.14, 1.98)
Houston, TX	17.70	27.97	15.45	1.81 (1.57, 2.09)
Indianapolis, IN	15.44	19.22	14.84	1.29 (1.04, 1.61)
Jacksonville, FL	15.14	17.20	15.02	1.15 (0.91, 1.44)
Los Angeles, CA	13.19	21.86	12.68	1.72 (1.49, 2.00)
Louisville, KY	13.22	18.75	12.85	1.46 (1.13, 1.89)
Memphis, TN	19.03	24.52	13.28	1.85 (1.47, 2.32)
Milwaukee, WI	17.49	23.23	14.47	1.61 (1.23, 2.09)
Nashville, TN	16.46	24.16	15.32	1.58 (1.23, 2.02)
New York, NY	13.09	16.78	13.07	1.28 (1.18, 1.39)
Oklahoma City, OK	13.94	22.76	12.94	1.76 (1.27, 2.44)
Philadelphia, PA	17.07	21.09	14.31	1.47 (1.27, 1.71)
Phoenix, AZ	12.73	21.41	12.97	1.65 (1.20, 2.28)
Portland, OR	13.90	—	14.05	—
San Antonio, TX	18.04	22.81	17.55	1.30 (0.98, 1.72)
San Diego, CA	12.36	15.87	13.51	1.17 (0.84, 1.65)
San Francisco, CA	12.47	20.98	12.38	1.69 (1.17, 2.46)
San Jose, CA	11.44	—	12.55	—
Seattle, WA	10.31	17.03	10.37	1.64 (1.06, 2.56)
Washington, DC	14.18	19.91	7.41	2.69 (1.96, 3.68)

— denotes data suppression as death counts were <20. CI refers to confidence interval.

**Figure 1:**
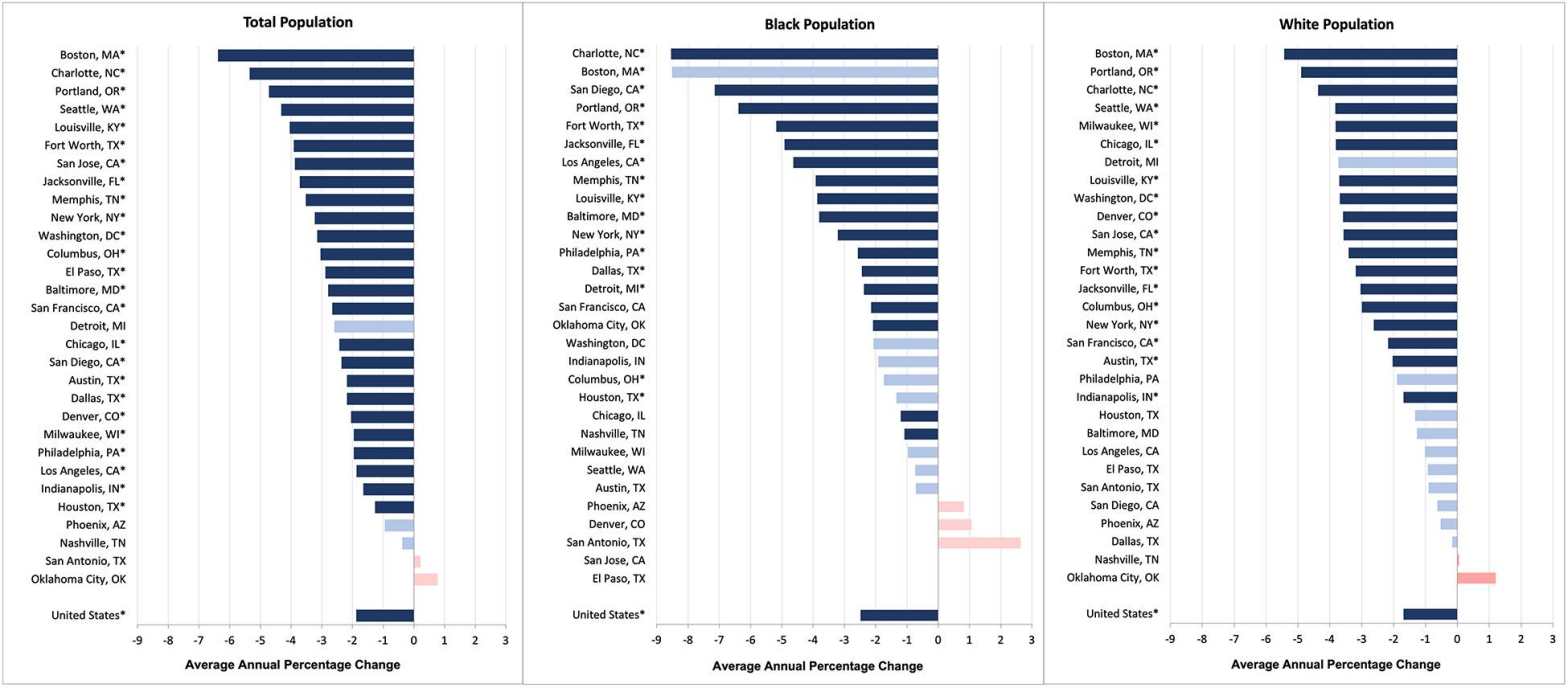
Average annual percentage change in colorectal cancer mortality rates for the total population, black population, and white population (2009–2019).^a^

The average annual US CRC mortality rate significantly decreased between 2009 and 2019 (AAPC: −1.88%; 95% CI: −1.98% to −1.78%; *P* < 0.001) (Figure 1). Twenty-five of the cities experienced significant declines in CRC mortality during this eleven-year study period. The AAPC for these cities ranged from −1.27% in Houston (95% CI: −1.63% to −0.91%; *P* < 0.001) to −6.38% in Boston (95% CI: −8.03% to −4.71%; *P* < 0.001). The CRC mortality rates for the following 5 cities remained stable over time: Phoenix, San Antonio, Detroit, Nashville, and Oklahoma City.

Sixteen cities showed a significant improvement in the average annual CRC mortality rate for their Black populations. The Black CRC mortality rate declined the most in Charlotte (−8.54%; 95% CI: −10.41% to −6.63%; *P* < 0.001) and the least in Houston (−1.34%; 95% CI: −2.49% to −0.19%; *P* = 0.023). Twelve cities did not experience any change in the Black CRC mortality rate. In terms of the White CRC mortality rate, 18 cities had significant declines. Of these, the improvement was greatest for Boston (AAPC = 5.43%; 95% CI: −7.74% to −1.12%; *P* < 0.001) and least for Indianapolis (AAPC = 1.69%; 95% CI: −2.36% to −1.01%; *P* = 0.001).

**Figure 2:**
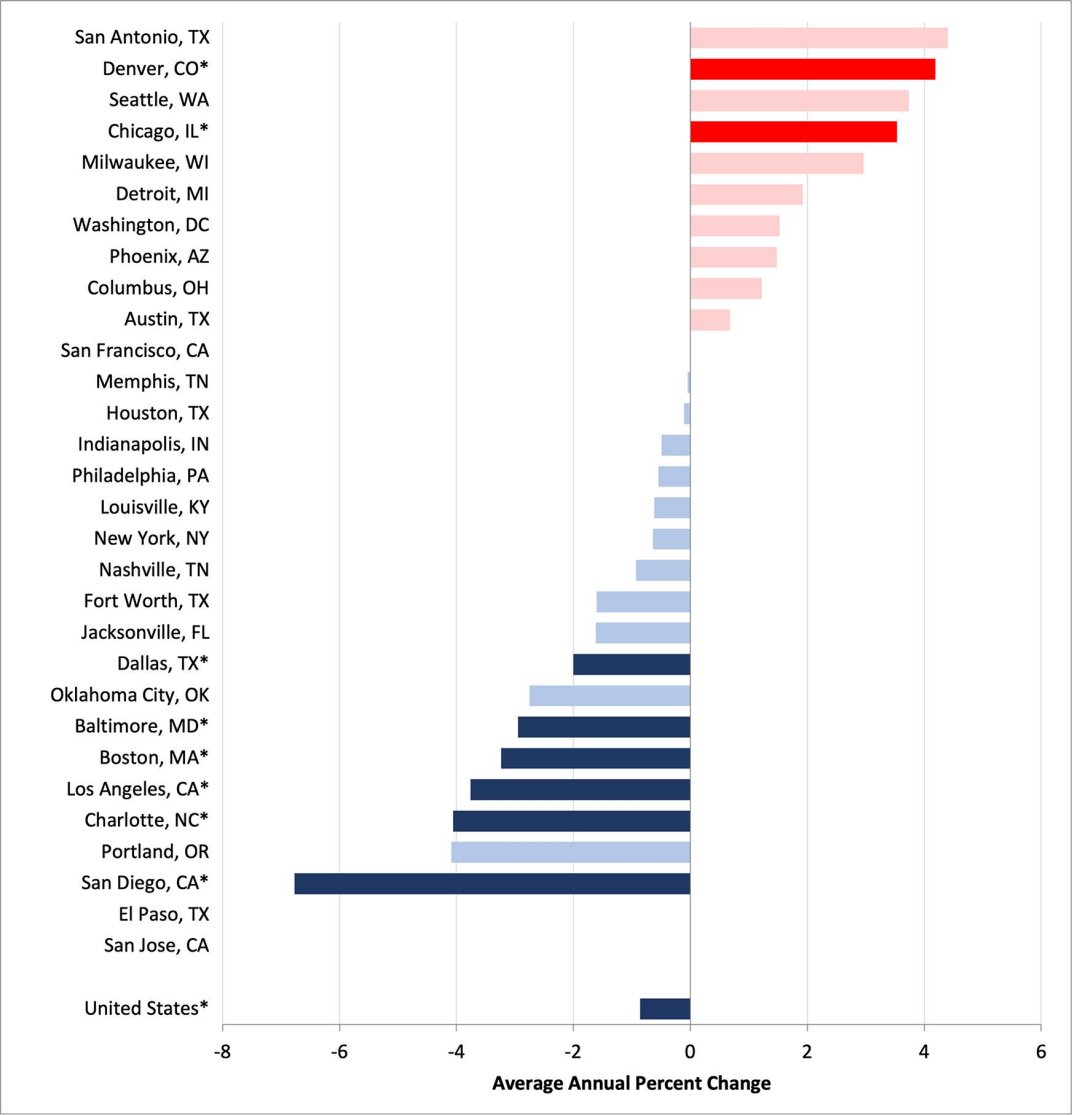
Average annual percentage change in Black:White colorectal cancer mortality rate ratios, 2009–2019.^a^

Nationally, the Black:White disparity in CRC mortality exhibited a statistically significant downtrend between 2009 and 2019 (Figure 2). More specifically, there was an almost 1% average annual decline in the Black:White RR (−0.86%; 95% CI, −1.12% to −0.61%; *P* < 0.001). Six cities also experienced statistically significant decreases in racial inequities: Boston, Baltimore, Dallas, Los Angeles, San Diego, and Charlotte. Among these cities, the AAPC in the RRs ranged from −6.77% in San Diego (95% CI −8.36% to −5.14%; *P* = 0.007) to −2.00% in Dallas (95% CI −3.65% to −0.34%; *P* = 0.025). Two cities experienced a statistically significant increase in racial inequity: Chicago (3.53%; 95% CI, 2.44%–3.53%, *P* < 0.001) and Denver (4.19%; 95% CI, 1.40%–7.05%, *P* = 0.01). The RRs for the remaining cities remained stable.

**Figure 3:**
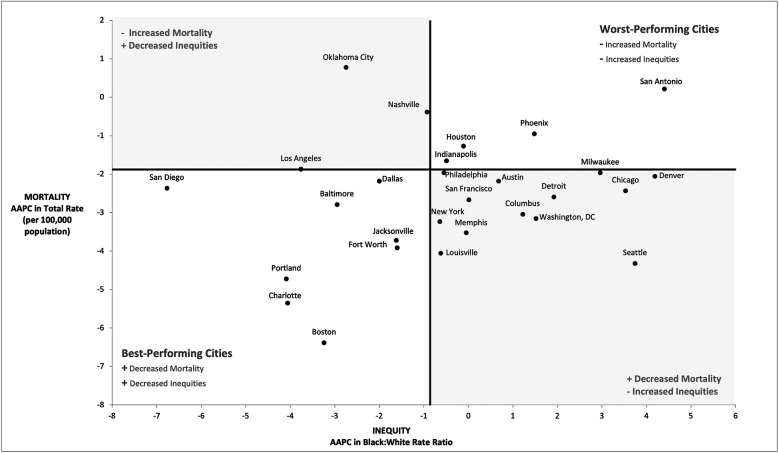
Annual average percentage (AAPC) change in colorectal cancer mortality rates and racial equity in rates (2008–2019).^a^

We plotted the 30 cities based on AAPC in CRC mortality and AAPC in the Black:White CRC rate ratio (Figure 3). We divided cities into quadrants using the national AAPC for total CRC mortality rate and the Black:White rate ratio. Eight cities (San Diego, Baltimore, Dallas, Portland, Fort Worth, Jacksonville, Charlotte and Boston) landed in the lower-left quadrant, which we labeled the best-performing cities. These cities performed better than the US in terms of decreasing CRC mortality and Black:White inequity. Conversely, the upper-right quadrant includes the worst-performing cities with both increasing CRC mortality rates and inequity as compared to the country as a whole. These cities include San Antonio, Houston, Indianapolis, and Phoenix. Most cities (*n* = 13) fell into the lower-right quadrant which represents those with a larger decrease in CRC mortality but higher increase in inequity. On the other hand, three cities (Oklahoma City, Los Angeles, and Nashville) performed worse than the country in terms of decreasing CRC mortality but fared better on reducing inequity.

## Discussion

This study analyzed CRC mortality rates and Black:White inequities nationally and across the 30 most populous cities. We made several important observations. First, CRC mortality rates significantly decreased nationally and across 25 cities. CRC mortality rates remained stable in the remaining 5 cities. Second, the magnitude of this decrease was generally higher for the Black vs. White populations, at both the national and city levels. Third, despite the steeper decline in the Black population CRC mortality rate, Black:White disparities remained stable in 20 cities. Nationally, and in 8 cities, these inequities decreased over time. These results indicate that the overall decline in CRC mortality is not distributed equally among geographic or racial lines, indicating persistent disparities and signaling the need for intervention. This is especially critical given a projected increase in CRC mortality rates in the coming decades (31).

Our findings align with the existing literature showing declining CRC mortality rates over the past decade at both national and subnational levels (2–4, 32). The improvement may be attributed to an increased frequency of screening examinations and advancements in both medical imaging and surgical techniques (10). Despite improvements in CRC mortality, Black:White disparities narrowly declined over time (10). Black patients are often unable to take advantage of improved CRC screening and treatment measures due to barriers stemming from structural racism, leading to persistent disparities (10). Neighborhood socioeconomic status plays a substantial role in perpetuating CRC inequities (33, 34). Black residents living in underserved neighborhoods have access to lower quality healthcare because of residential segregation tracing back to redlining (35, 36). Black patients are also more likely to face treatment delays compared to their White counterparts, which may be exacerbated by transportation barriers (37, 38). These findings indicate the need for a proactive approach that identifies and addresses the needs of minoritized individuals diagnosed with CRC earlier on, with a focus on under-resourced communities.

Differences in access to screening also perpetuate disparities in Black:White CRC mortality (39). CRC screening rates are notably lower among Black individuals compared to White individuals in the US (39). Differences in screening rates may be attributed to a variety of causes, such as lack of CRC symptoms, cost, and lack of physician recommendation (39). A study assessing adherence to CRC guidelines among Black patients that 40% of non-adherent individuals reported to have never received recommendation from their provider to screen for CRC, indicating that some Black patients receive different clinical guidance than their White counterparts (40). Given recent guideline changes recommending earlier screening for younger populations, future research could examine city-level CRC mortality stratified by sex and age as well (41).

Disparities have been identified at the state and county level across the nation (10, 42, 43). By identifying trends in geographic disparities at an even more local level, we intend to empower local governments to create specific, targeted inventions that promote health equity. These interventions should aim to reduce barriers to CRC screening to promote earlier diagnosis and treatment, which should reduce mortality. Successful initiatives in Delaware and New York City involved a combination of reducing screening barriers, increasing health education, and providing patient navigation (22, 44). The Delaware Cancer Treatment program led to an increase in CRC screening rates for Black patients from 48% to 74%, equal to the rate among White residents (22). This also led to more early-stage diagnoses, and the CRC mortality rate among Black patients declined by 42%, indicating the utility of screening in reducing CRC mortality and the efficacy of focused, place-based initiatives (22, 44).

The Affordable Care Act (ACA) may have implications for CRC screening and mortality by eliminating out-of-pocket screening costs. One study found that Medicaid expansion in Kentucky led to a significant increase in CRC screening for patients with Medicaid and a decrease in CRC mortality in the short-term (45). Other studies assessing CRC screening have similarly identified increases in screening rates and decreases in CRC mortality after the implementation of ACA (46, 47). This is a promising finding, indicating the need for more data on the impact of ACA on CRC mortality in the long-term.

A primary strength of our study is that it provides data on CRC mortality at a city level over time. This allows us to assess how CRC mortality is improving or worsening over time across geographic areas. We also analyze differences in CRC mortality trends among Black and White populations to evaluate changes in racial disparities. Identifying cities that demonstrate progress toward inequity may help provide potential solutions for eliminating CRC mortality inequity across a wider range of cities across the US. Conversely, recognizing cities that endure persistent or worsening racial inequities prompts the exploration of factors that prohibit progress.

This study is also subject to limitations. Our analysis only evaluated mortality differences between Black and White populations. We focused on these groups due to the large degree of inequity between these populations and to emphasize racial disparities in CRC care. Other racial and ethnic groups, such as Asian and Hispanic/Latinx populations, see fewer CRC deaths than the Black or White populations. Despite this, it is important to highlight inequities among all minoritized populations. Further studies should evaluate differences among other racial and ethnic groups to complete our understanding of CRC disparities and to guide more effective public health interventions. Another limitation relates to geography. In some cases, counties were used as proxies for cities. These counties may include suburban populations which could differ from urban populations in access to CRC screening and care. Demographic changes during the study period may also have occurred and thus impacted the rates observed in some cities. Future, longer-term analyses account for these changes, particularly as we assessed CRC mortality trends post-pandemic. Another potential limitation is the definition used to define CRC mortality. The use of ICD-9 codes is not consistent across platforms (15, 23, 24). The Healthy People Initiative, with an overarching goal of achieving health equity, includes anal cancer in their CRC counts, and we follow this definition in our analyses. This may cause slight differences in our calculated CRC mortality rates, but since anal cancer accounts for a very small percentage of all CRC diagnoses, these differences are minimal.

## Conclusion

Despite advancements in cancer care, CRC remains a leading cause of cancer deaths in the US. While on a national level, Black:White CRC mortality disparities are decreasing over time, these disparities persist in the majority of our most populous cities. Importantly, the current analyses utilize data obtained prior to the COVID-19 pandemic. The pandemic initially disrupted cancer screenings through the delay of non-urgent medical procedures, leading to an 85% decrease in CRC screenings between April 2019 and April 2020 (19). Delayed and cancelled CRC screenings can lead to delays in diagnosis and treatment, worsening existing CRC disparities (48). It is important to replicate this study using post-pandemic data to fully understand changes in CRC mortality disparities over time. Analyzing these inequities at a city level serves to empower local governments and public health officials to enact legislation and policies that ensure equitable access to cancer care.

## Data Availability

Public use mortality datasets can be found at https://www.cdc.gov/nchs/nvss/mortality_public_use_data.htm or accessed at https://wonder.cdc.gov/. The datasets analyzed during the current study are restricted-use vital statistics data obtained from the National Vital Statistics System (NCHS) via a data use agreement. Data are available from the authors upon reasonable request and with permission from NCHS.
